# Mechanosensitive Adaptation of E-Cadherin Turnover across *adherens* Junctions

**DOI:** 10.1371/journal.pone.0128281

**Published:** 2015-06-05

**Authors:** Simon de Beco, Jean-Baptiste Perney, Sylvie Coscoy, François Amblard

**Affiliations:** 1 Laboratoire de Physico-Chimie, Centre de Recherche, Institut Curie, Paris, France; 2 Centre National de la Recherche Scientifique, Unité Mixte de Recherche 168, Paris, France; 3 Université Pierre et Marie Curie, Paris, France; Northwestern University Feinberg School of Medicine, UNITED STATES

## Abstract

In the natural and technological world, multi-agent systems strongly depend on how the interactions are ruled between their individual components, and the proper control of time-scales and synchronization is a key issue. This certainly applies to living tissues when multicellular assemblies such as epithelial cells achieve complex morphogenetic processes. In epithelia, because cells are known to individually generate actomyosin contractile stress, each individual intercellular adhesive junction line is subjected to the opposed stresses independently generated by its two partner cells. Contact lines should thus move unless their two partner cells mechanically match. The geometric homeostasis of mature epithelia observed at short enough time-scale thus raises the problem to understand how cells, if considered as noisy individual actuators, do adapt across individual intercellular contacts to locally balance their time-average contractile stress. Structural components of *adherens* junctions, cytoskeleton (F-actin) and homophilic bonds (E-cadherin) are quickly renewed at steady-state. These turnovers, if they depend on forces exerted at contacts, may play a key role in the mechanical adaptation of epithelia. Here we focus on E-cadherin as a force transducer, and we study the local regulation and the mechanosensitivity of its turnover in junctions. We show that E-cadherin turnover rates match remarkably well on either side of mature intercellular contacts, despite the fact that they exhibit large fluctuations in time and variations from one junction to another. Using local mechanical and biochemical perturbations, we find faster turnover rates with increased tension, and asymmetric rates at unbalanced junctions. Together, the observations that E-cadherin turnover, and its local symmetry or asymmetry at each side of the junction, are mechanosensitive, support the hypothesis that E-cadherin turnover could be involved in mechanical homeostasis of epithelia.

## Introduction

How complex systems self-organize is known to strongly depend on the dynamic properties of their individual components, but also on the time-line of the interactions between them. In biological tissues such as epithelia, dynamic properties and a high degree of intercellular coordination are involved in morphogenetic movements such as cell patterning [1], intercalation [2], tubulogenesis [3], and collective cell migrations in general [4, 5]. Numerous investigations of these phenomena in the past decade have led to strong evidence that stress within and between epithelial cells is essentially produced by actomyosin-based contraction, and primarily transmitted by epithelial cadherin (E-cadherin) bonds across *adherens* junctions (AJs). In addition, E-cadherin bonds are not mere transducers passively opposing detachment forces, but they regulate in a mechanosensitive way the assembly and the activity of the contraction-adhesion machinery [6].

In mature homeostatic epithelia, the cytoskeletal and adhesion architectures remain highly dynamic [[7–11, 12]. Various biochemical turnover processes are involved and dissipate metabolic energy with a range of turnover time scales of minutes or tens of seconds. At these time-scales however, the epithelial geometry is stable: indeed, junction are quasi immobile at a time-scale (10 minutes) where F-actin cytoskeleton is entirely renewed, and at longer time-scales exhibit weak non-monotonous movements restricting their global displacements ([13–15], S1 Fig and S2 Movie). The question therefore stands out of the physiological meaning of this sustained energy dissipation. A simple hypothesis is that the observed turnover of AJs could be necessary to mechanically adapt adjacent cells to each other and stabilize a steady-state multicellular geometry. In that case, it would be expected that turnover in AJ should be mechanosensitive (in order to adapt to applied forces) and tightly coordinated on each side of the junction. This mechanosensitivity would fit with the observation that the dynamics of E-cadherin is modulated by the local organization of cytoskeleton and its contractile activity refref étof[16–17], but it has not been tested directly so farrefref.

In this paper, we focus on the temporal properties of E-cadherin homophilic bonds between adjacent epithelial cells, in steady-state junctions and in junctions submitted to mechanical perturbations. We test the hypothesis that E-cadherin turnover is mechanosensitive and locally coordinated. In a previous work, we showed by FRAP that E-cadherin turns over at mature AJs [11–12] as a first-order rate limited process with an average residence time symsym τ_res_ = 4 min in MDCK cells. The nature of E-cadherin movement (first-order rate exchange and no membrane diffusion) was demonstrated by spatial analysis of two-photon FRAP experiments. A central argument of this analysis was the absence of widening in fluorescence profiles during FRAP relaxation. Moreover, using inhibiting drugs (Dynasore, MiTMAB), we demonstrated that endocytosis is the rate limiting step of E-cadherin turnover in confluent monolayers of MDCK cells [11]. In other cell lines, endocytosis may still play a role but other first-order rate-limiting mechanisms might be involved [18–19]. Despite the key role of endocytosis, its exact contribution as a determinant of E-cadherin residence time is not the central question of the present work, and dynamics will only be reported in term of turnover rates or residence times. However we systematically checked that the observed dynamics correspond to first-order reaction and not membrane diffusion even in the case of mechanical stimulation.

The questions addressed here are the following: To what extent are E-cadherin turnover rates matched between adjacent cells, and dependent on the intercellular tension? Regardless of the molecular determinants and mechanistic underpinnings of E-cadherin turnover, these questions will be addressed *in vitro* with the MDCK cellular model, using two-color two-photon FRAP and micromanipulations. In this paper, we show that turnover rates vary considerably in space and fluctuate in time, but that they match remarkably well across individual cell-cell contact lines. Our results provide the direct evidence that the rate of E-cadherin turnover in a cell is locally regulated in intercellular contact, in synchrony with its partner cells and in a mechanosensitive manner.

## Results

### E-cadherin turnover rates varies in space and fluctuate in time

**Fig 1:**
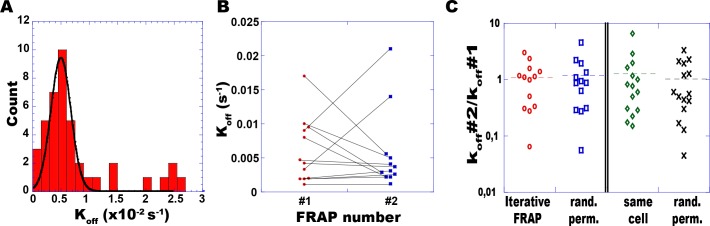
E-cadherin turnover rates vary in space and fluctuate in time. (A) Distribution of turnover rates (k_off_) of E-cadherin-GFP measured by FRAP at diffraction-limited spots at different junctions across a confluent layer of MDCK cells. For each junction (N = 41) the individual fluorescence recovery curve was fit with an exponential relaxation. The solid curve shows a gaussian fit (average rate 4.8 10^-3^ s^-1^ and standard deviation 2.5 10^–3^ s^-1^) of the distribution of the 34 non-outlying values. (B) At each single spot, the turnover rate was measured twice (#1 and #2) with a 45 minutes time interval. (C) For each spot, the temporal variation of the turnover rate is assessed by the ratio of the successive measurements k_off_#2/ k_off_#1 (iterative FRAP, left panel, red circles). This ensemble of ratios was compared to the similar ensemble obtained after a random permutation of the rates within the first set (#1) (rand. perm., blue squares). Rates were also compared within matched pairs of spots belonging to two different AJs of the same cell (right panel): ratios for matched pairs (green diamonds) were compared to mismatched pairs (random permutations of values, black crosses). No significant correlations were found between rates, neither in time when sampled across a 45 minutes interval, or in space within single cells.

In order to study the spatial dispersion of E-cadherin dynamics, individual E-cadherin turnover rates (k_off_ = 1/ symsym τ_res_) were locally measured at diffraction-limited spots belonging to distinct cell-cell contacts, in confluent layers of MDCK cells. These experiments are similar to our previous paper about E-cadherin dynamics (11), with details and examples shown in S2 Fig, but here we focused on individual measurements and not on mean curves. Rates were found to be approximately scattered across one and a half order of magnitude, with a mean value k_off_ = 4.8 10^–3^ ± 0.4 10^–3^ s^-1^ (Fig 1A), similar to our previous result (k_off_ = 4.3 10^–3^ ± 1.1 10^–3^ s^-1^) [11].

To examine to what extent these variations correspond to static heterogeneities among epithelial cells, rates were assessed over a set of spots for time pairs, i.e. at two time points 45 minutes apart. During this time interval, measured rates exhibited large variations of up to 10-fold at single spots (Fig 1B), with no visible correlation between the values found within time pairs (Fig 1C left panel, Table 1). In fact, the distribution of ratios k_off_#2/k_off_#1 in each spot is scattered over more than an order of magnitude, and similar to a situation where the pairs of k_off_ are randomly permutated. This strongly suggests that the dispersion of E-cadherin turnover rates is not static, but rather reflects spatially independent temporal fluctuations at individual AJs. In addition, virtually no correlation was found between rates measured simultaneously at 2 spots belonging to distinct contacts of the same cell (Fig 1C right panel, Table 1). This strongly suggests that E-cadherin turnovers are not globally controlled at the level of the individual cell. However, the single-color FRAP method used so-far yield a single rate from possibly distinct behaviors in each partner cell.

**Table 1:** Spearman rank correlation analysis for pairs of E-cadherin turnover rates.

	Spearman coefficient ρ	ρ random permutations
Same junction, successive FRAP	0.71	0.48
Same cell, different junctions	0.28	0.21
Same junction, dual color FRAP	0.94	0.26

E-cadherin turnover rates were measured by FRAP as follows: iterative pairs were obtained by measuring E-cadherin turnover rates in a same spot at two time points 45 minutes apart (line 1). Single-cell pairs were obtained by measuring rates simultaneously at two distinct junctions of a same cell (line 2). Single-junction pairs were assessed by dual-color FRAP on E-cadherin from two neighbor cells in a same junction (line 3). Correlations within pairs (between k_off_#2 and k_off_#1) were estimated by computing the Spearman coefficient symsymρ. As a comparison to evaluate the strength of the correlation, this result is compared to a randomized situation, obtained by a random permutation within one set of rates (k_off_#2). Significant correlations (p<0.05) are shown in boldface.

### Turnover rates match across unperturbed mature intercellular contacts

**Fig 2:**
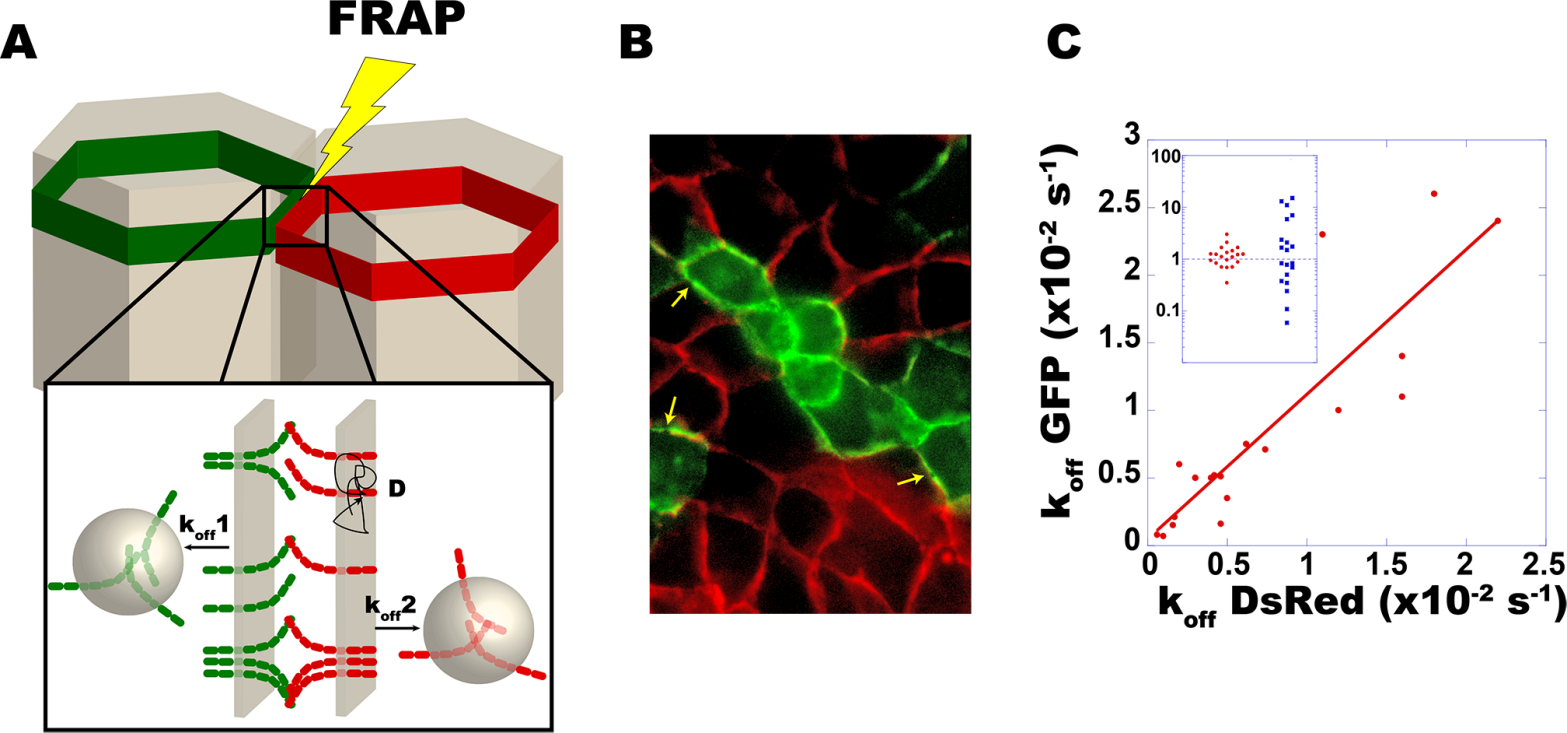
E-cadherin endocytosis rates match across individual unperturbed AJs. Endocytosis rates of E-cadherin were measured using dual-color FRAP on mature heterochromatic AJs between MDCK cells expressing either E-cadherin-GFP or E-cadherin-DsRed (A, arrows in B). Kinetics were recorded simultaneously but resolved separately on each side of a same junction (A insert). Each measurement led to a pair of rates shown as a point in a biparametric logarithmic plot (k_off_^DsRed^, k_off_^GFP^) (C). A linear fit gives a rate ratio k_off_^GFP^/k_off_^DsRed^ = 1.07 (red line, R = 0.88). The distribution of ratios of rate pairs k_off_^GFP^ /k_off_^DsRed^ (insert, red dots) is compared to what is obtained following a random permutation with the set of k_off_^DsRed^ values (blue squares).

In order to investigate how E-cadherin turnover is coordinated between adjacent individual cells, we sought to separately resolve the dynamics of E-cadherin molecules across individual junctions, and developed a 2-colour FRAP assay (Fig 2A). Using two-photon excitation at a single wave-length for two distinct fluorophores, spatial coincidence problems were avoided, and the dynamics of E-cadherin could be comparatively assessed within homophilic complexes at a microscopic resolution. A chromatically chimeric epithelium was produced (Fig 2B), by mixing MDCK cells expressing E-cadherin exclusively fused to GFP or DsRed. These cells did mix well and produced a *bona fide* epithelial culture. At confluence, these cells produced homo- and hetero-chromatic AJs with no detectable difference, in which the dynamics of E-cadherin-DsRed and E-cadherin-GFP were in average identical. In hetero-chromatic AJs, dual color FRAP was achieved simultaneously using a femtosecond laser oscillator tuned at 890 nm to optimally balance bleaching rates. Green and red fluorescence signals were imaged simultaneously (Fig 2B), and recovery curves were independently fitted by a first-order relaxation to obtain a pair of rates.

Strikingly, rates assessed on both sides of individual heterochromatic contacts are remarkably close with a strong statistical significance (Fig 2C, Spearman ρ = 0.94). Indeed, the ratios between *trans*-junction pairs k_off_GFP/k_off_DsRed (red dots in the upper insert of Fig 2C) show a much narrower distribution than randomly permutated pairs (blue squares). In other words, while E-cadherin turnover rates do fluctuate with time and vary in space, they remain matched across the junction in a highly local manner, with stronger correlations than between cadherins at distinct contacts of the same cell (Table 1). This strongly suggests that E-cadherin turnover rate is regulated very dynamically and locally, with a length scale in the micrometer range, in a way that is sensitive to local properties of the neighbor cell.

### Turnover rates symmetrically increase with increased contact stress

**Fig 3:**
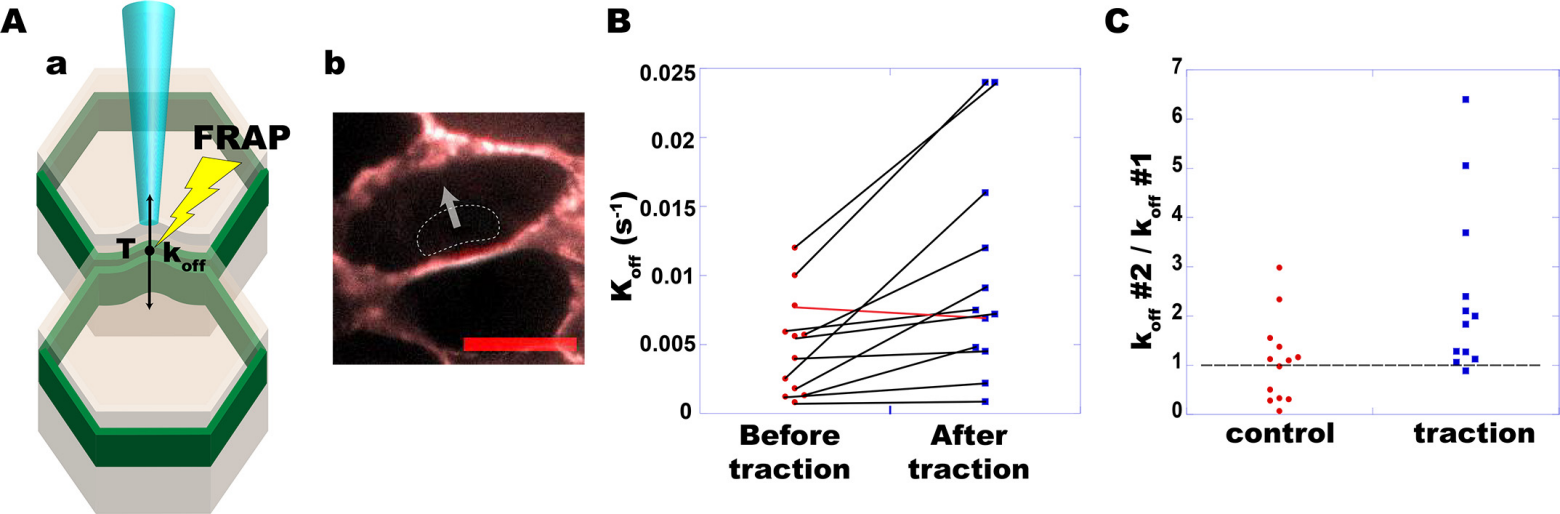
E-cadherin turnover rates increase with intercellular tension. (A-a) A perpendicular force was applied on individual junctions by pulling on the apical surface with a glass micro-pipette. The junctions were then clamped at a stable position approximately 0.5 μm away from the initial one. (A-b) Typical overlay image of a cell before (gray) and after traction (red), with the micro-pipette (dashed line) and the force vector (arrow). Scale bar: 10 μm. (B) FRAP was achieved on MDCK cells expressing E-cadherin-GFP, giving for each AJ a pair of rates (k_off_) before (red dots) and after (blue squares) traction (N = 12). (C) For each rate pair, the ratio k_off "after"_/ k_off "before"_ is shown (blue squares), and compared to control experiments without traction (red dots). Turnover rates increased after tension was applied in all (N = 11) but one case (red line in B).

Following our working hypothesis that E-cadherin turnover is involved in balancing intercellular stress between adjacent cells, we then questioned whether the turnover rate of E-cadherin is mechanosensitive. To investigate this possibility, cell-cell junctions were perpendicularly stressed by pulling on the apical surface with a micropipette while assessing the turnover rate. More precisely two situations could be compared: (1) No artificial traction was applied to cell-cell junctions; (2) after exerting a perpendicular traction and clamping the contact line approximately 0.5 μm away from its initial position. A new steady-state position is rapidly obtained, by maintaining a constant position of the micropipette (Fig 3A). In the latter situation, the apical surface transmits a perpendicular stress increase to the contact line. Indeed, that line is observed to slowly relax (approximately 10–15 minutes) to its initial position when the micropipette is removed (S3 Fig), showing that the elastic stress is maintained throughout the duration of the clamp.

Using dual-color FRAP, we could not detect any significant mismatch of E-cadherin turnover rates across stably clamped cell-cell contacts (S5 Fig), and we therefore assumed that the bilateral average rate measured in single-color FRAP experiments reflects the common value of both rates k_off_ DsRed and k_off_ GFP. Consequently, the effect of tension was further assessed in single-color FRAP experiments, before and after the micropipette traction. This bilateral average did increase for all junctions but one (red line), and the average fold-increase over all junctions equals 2.4, while no trend was seen in the unperturbed situation (Fig 3). Because rates were not individually measured, a slight rate asymmetry cannot be directly ruled out across clamped contacts (S5 Fig). Nevertheless, the main conclusion holds that both rates do increase, and the argument is mainly based on the amplitude of the observed 2.4 fold-increase of the average (Fig 3C). Indeed, if we assume a moderate asymmetry, such as DsRed / k_off_GFP = 1.4 after traction (median in S5 Fig), we obtain that k_off_ DsRed and k_off_ GFP should increase by a factor 2.8 and 2 respectively. And if we assume a larger asymmetry, with DsRed / k_off_GFP = 1.8 after traction (the tail of the distribution observed in S5 Fig), we obtain that k_off_ DsRed and k_off_ GFP should increase by a factor 3.1 and 1.7 respectively. In other words, even if turnover rates were not exactly symmetrical, the conclusion that they both increase still holds. Turnover involves two distinct fluxes, with molecules being taken away from the observation volume at the membrane, and others arriving into that same volume. Increased recovery primarily means a faster flux of unbleached molecules toward the observation volume, and this faster input flux is balanced by the output flux at steady-steate. Should it not be balanced by an increased output flux, and the output flux remain constant, the concentration of E-cadherin at the membrane should then increase. We do not observe such an concentration increase, but rather a slight decrease (5%) after applying the tension (S4 Fig). This data thus suggest that both fluxes increase, leading to an overall increase of the turnover. This faster turnover could not be attributed to increased diffusion of E-cadherin either, since we could not detect any widening of fluorescence profiles during the recovery process (S6 Fig, see also [11] for more details on this spatial analysis). Instead we detected a decrease of the width of fluorescent profiles, which could be attributed to heterogeneous exchange rates along the junctions. Indeed, the extra tension provided by pulling junctions with pipettes is probably higher at the junction center where the pipette is applied compared to its sides. In case of mechanosensitive turnover rate, it would be consistent with heterogeneous rates and the decrease of width in FRAP profiles that we observed. This strongly suggests that E-cadherin turnover is a mechanosensitive process, which speeds up when the stress on AJs is increased above its basal level.

### Asymmetric turnover rates are induced by asymmetric contraction

**Fig 4:**
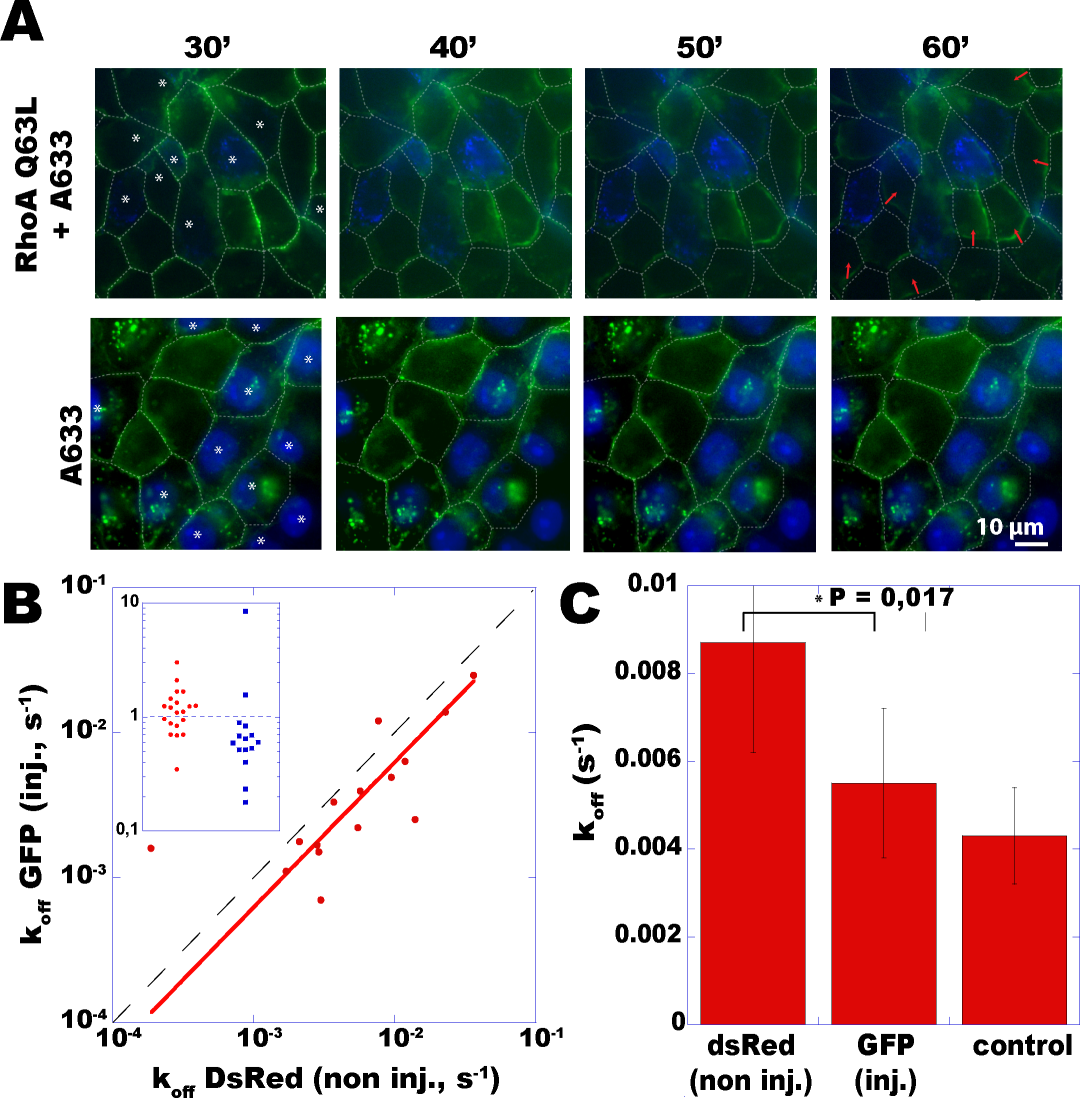
Induced force asymmetry triggers asymmetric turnover rates across *adherens* junctions. (A) MDCK-Ecadh-GFP cells exhibit an apical constriction when microinjected with RhoA-Q63L + Alexa633 (upper row), but not with Alexa633 alone (bottom row). Injected cells show nuclear staining (displayed in blue, as well as with white stars in the first time point). Red arrows indicate junction motions relative to their position at 30 minutes post-injection (white dashed lines). (B) E-cadherin turnover rates were measured by dual-color FRAP across a set of N = 15 junctions, each taken between one RhoA-injected Ecadh-GFP-expressing and a neighbor non-injected Ecadh-DsRed-expressing MDCK cell. The curve k_off_^inj. cell^ = f(k_off_^non inj. cell^) (red dots) was fitted by a linear regression (red line, coefficient R = 0.91, k_off_^injected^/k_off_^non-injected^ = 0.65). The ratio value r = 1 is shown by the dashed diagonal. Insert in (B): the set of ratios k_off_^injected^/k_off_^non-injected^ (blue squares) significantly shifts below 1, as compared to ratios between pairs of non-injected cells (red dots). (C) Comparison of rates between injected, non-injected, and (N = 20) control cells, showing a significant difference (*) (paired Student’s *t* test).

Above observations show that E-cadherin rates locally match at stable contacts. One can further question whether these rates should mismatch at mechanically asymmetric contacts, where a persisting force asymmetry leads to a purely convective, monotonous movement. Individual contacts were made mechanically asymmetric by micro-injecting a constitutively active form of RhoA expected to trigger myosin-II dependent contraction and apical tension [20]. Indeed, the injection of 0.2 mg/mL RhoAQ63L coupled to Alexa-633 resulted in apical contraction of MDCK cells (Fig 4A, red arrows). For more than an hour after injection, the apical portion of the contact of injected cells drifted monotonously toward the shrinking cell. Although the detailed biochemistry and mechanics of this persistent drift is complex, it did reflect a persistent mechanical asymmetry at the level of individual intercellular contacts.

Across such drifting contact lines, dual color FRAP revealed locally asymmetric E-cadherin turnover rates (Fig 4B). Noteworthy, rates were still linearly related (with a linear regression coefficient R = 0.91) by a ratio significantly different from 1: k_off_^contracting^/k_off_^opposed^ = 0.65 ±0.09 (Fig 4B, insert). Rates still corresponded to a first order space-independent recovery, with a time-independent width of the spatial fluorescence profile during recovery (S7 Fig), thus ruling out the possibility of enhanced membrane diffusion of E-cadherin. In average, rates were found unchanged in the injected (contracting) cells (Fig 4C), but significantly increased in their adhesive partners. These facts, together with the observed rate increase induced by an increased tension, could be interpreted as follows: (**a**) drifting contacts could be subjected to a larger tension due to a contraction increase in the injected partner, thus (**b**) inducing a faster turnover in the non injected partner, while (**c**) this expected rate increase is somehow antagonized in the injected partner by a stabilizing effect of RhoA, as already reported in the literature. Regardless of the molecular basis, our results in symmetric and asymmmetric situations further support the notion of a direct relationship between how fast E-cadherins turn over inside a cell at a given contact, and how much stress it exerts on its partner across that contact.

## Discussion

The stabilization of epithelial junctions is a very complex problem of geometry, mechanics and molecular regulations. Adhesive bonds do not generate stress *per se* but transmit it, and the observed E-cadherin turnover implies that stress transmission is collectively sustained by a number of transient intercellular bonds. Therefore, E-cadherin turnover and its rate likely impact on the detailed balance of forces at intercellular contacts, whether active contraction or friction. Indeed this is not the only known determinant of mechanical regulation in *adherens* junction. The conformation of α-catenin has been shown to change with varying tension, therefore functioning as a molecular switch able to recruit vinculin in a force-dependent fashion. Vinculin itself has been shown to potentiate the mechanosensitive functions of the whole cadherin-catenin complex, by recruiting myosin and thereby reinforcing the strength of the junctions [21–25]. Force-dependent conformational changes in α-catenin have also been shown to stabilize the binding of actin to the cadherin-catenin complex [26]. However, the role of E-cadherin turnover in the regulation of transmitted forces has not been addressed so far.

The detailed mesoscopic organization of E-cadherin bonds remains elusive with *cis*- and *trans*-bonds, while each of them probably experiences a different tension on their intracellular and extracellular regions [27]refr. However, intercellular contacts are subjected to constitutive contractile stress [28] generated by the noisy activity of the acto-myosin networks in each partner cell. We study here how cells interact by analyzing some temporal properties of the intercellular contacts. Our first important finding is that the rate at which E-cadherin turns over (i.e. a few minutes here) strongly fluctuates in time and space. Intercellular contacts appear to be dynamically autonomous at a very local spatial scale, but to individually obey characteristic symmetry rules: E-cadherin turnover is isochronous across junctions, and this symmetry correlates with the quasi-immobility of junctions at a time-scale of 10–20 minutes, which reflects a mechanical stability (see S1 Fig).

The second finding, that this turnover rate is mechanosensitive, is congruent with previous reports of the mechanosensitive behavior of AJs components [29] [25, 30–32]refref. These studies nicely showed that proteins anchoring cadherins to the cytoskeleton, including α-catenin and vinculin, are involved in adaptation of *adherens* junctions to varying tensions. It was demonstrated that these proteins participate in reinforcing the junctions and strengthening adhesion in response to increasing stress. However, while the influence of intercellular tension on E-cadherin engagement was so far mostly reported so far as a positive feedback leading to the notion of force-induced reinforcement of adhesion, our observation also suggests the existence of a negative feedback by which E-cadherin turnover speeds up for larger stresses. Both mechanisms might collaborate, on the one hand to initiate intercellular adhesion and reach full epithelial differentiation, and on the other hand to limit the transmission of excessive stress at contacts.

Indeed these results are not due to diffusion of E-cadherin but to actual turnover rates. We have previously demonstrated that E-cadherin at AJs do not diffuse but are turned over within minutes, endocytosis being the rate-limiting process [11]. Here we could verify that increased E-cadherin dynamics in mechanically perturbed junctions were in fact the result of increased turnover rates, since no diffusion could be observed through spatial analysis of FRAP recovery profiles. Hence, these data suggest that the mechanisms regulating E-cadherin trafficking between the membrane and the cytoplasm themselves are mechanosensistive. This could be due to facilitation of bond breakage or regulation of the endocytosis/exocytosis machinery.

Although the primary actuator is the actomyosin cortex with its multiple regulatory mechanisms [33], E-cadherin adhesive bonds control how much of the contractile stress is transmitted between adjacent cells. The present work suggests that the rate of E-cadherin turnover could be a determinant of stress transmission and consequently provide a key time-scale of that dynamic control. Further work is needed to elucidate how the observed mechanosensitivity and symmetry rules contribute to the mechanical homeostasis of epithelia as a collective process.

## Materials and Methods

### Cell culture

Cells were grown in DMEM supplemented with 10% FCS and 0.4 mg/mL geneticin (Invitrogen). MDCK cells stably expressing E-Cadherin-GFP or E-Cadherin-DsRed were gifts from W. J. Nelson. (Stanford University, Stanford CA).

### Two-photon and two-colours FRAP

Single-colour two-photon FRAP was performed as previously described [9, 11]. In brief, cells were observed on glass coverslips ~ 12–24 h after confluency, in DMEM-FCS medium supplemented with 10 mM Hepes at 37°C on an IX71 inverted microscope (Olympus, Tokyo) with a high numerical aperture objective (63x oil immersion, NA = 1.25, PlanNeofluar, Zeiss, Jena).

Two-photon photobleaching was performed with a femto-second laser tuned at 878 nm, controlled by galvanometric mirrors and driven by MetaMorph. The measured excitation Point-Spread Function (PSF) is an ellipsoid with a 0.5 μm diameter in the focal plane and a 1.5 μm extension along the optical axis. Fluorescence recovery was spatially resolved under one-photon excitation by fast 3D wide field videomicroscopy, using a DG-4 monochromator (Sutter, Raleigh, NC-USA), a Coolsnap HQ CCD camera (Roper Scientific, Tucson, AZ-USA), and a PiFoc piezo-driven objective actuator (Physik Instrumente, Karlsruhe, Germany). FRAP experiments were performed as follows. One point in a junction was photobleached for 200 ms with a 30 mW average power excitation intensity. Stacks of 3–20 images, depending on experiments, with a 0.3 μm vertical spacing were acquired before and after photobleaching with a time interval Δt ranging from 100 ms to 30 s. See S1 Movie as an example.

For dual-colour FRAP experiments, the following changes were made: both fluorophores were bleached inside diffraction-limited volumes with a femto-second laser tuned at 890 nm (Mai-Tai, Spectra-Physics, Mountain View). Cells were bleached and observed with a high numerical aperture (NA = 1.42) objective at 60X (PlanApoN, Olympus, Japan). GFP and DsRed signals were simultaneous recorded using a DualView system (Optical insight, LLC, Santa Fe, USA), with a chromatic split at 565 nm. Bleaching was achieved using 200ms long pulses, with a 40mW average power at the focus (70mW at the back aperture). Image rate was 1/12s.

### Analysis and interpretation of the FRAP data

As already described in [11], image stack analysis was performed using a home-made program (code available upon request) using Matlab (Mathworks, MA-USA). Line intensity profiles were manually followed as a function of time with the possibility of correcting for possible transversal motions. These profiles were low-pass filtered over the waist of the PSF. Unwanted photobleaching during the recovery (at least 5–10% in 30 s as assessed on fixed cells) was corrected for by normalizing intensity histograms at each time point on the 3D stack. Fluorescence relaxation was analyzed either spatially or at a single point. The curves were normalized in such a way that 100% corresponded to the pre-bleach intensity and the 0% level corresponded to the level reached upon maximal bleaching depth, i.e. reached under equivalent conditions with paraformaldehyde-fixed GFP-expressing cells. FRAP data were analyzed using a reaction-limited model, based on results obtained previously: we systematically checked that there was no widening of fluorescence profile during recovery, that would be indicative from membrane diffusion. For dual-colour FRAP experiments, fluorescence signals in each image were analyzed simultanously, so as to make sure the signal corresponded to identical pixels in each image.

Interpretation of FRAP data in terms of an homogeneous first-order relaxation requires the assumption of a stationary state, i.e. a constant density of E-cadherin-GFP molecules at intercellular contacts, at least at time-scales significantly longer (30min to 1 hour) than the turnover times inferred from fits. In the present paper, we checked in all conditions that this stationary-state assumption was realistic.

### Microinjections, mechanical perturbations, and junction motion analysis

An injection mix was prepared in an iso-osmolar solution (Potassium Gluconate 130mM, NaCL 2mM, Hepes 20mM, MgCL2 4mM, Na2ATP 4mM, NaGTP 0.4mM, EGTA 1mM) containing constitutively active RhoA (RhoA Q63L, Cytoskeleton Inc, 0.2mg/mL) and Alexa 633 (0.04 mg/mL, Invitrogen). This solution was microinjected with glass micropipettes (injection radius: 0.5 μm) at low pressure (50 to 100 hPa) during 1s, using a Femtojet system (Eppendorf). FRAP was performed 30 minutes after injection. Direct mechanical perturbation was also carried out using glass micropipettes (5 μm radius). The pipette was put in contact with the apical plasma membrane and pulled gently using a micro-manipulator (Sutter Instruments MP-285, Novato CA, USA), in the planar direction perpendicular to the junction of interest, so as to move it and clamp it 0.5 μm away from its initial position. FRAP was performed immediately.

## Supporting Information

S1 Fig Cell junctions fluctuate in position. (A) 2 examples of cell junction trajectories over 20 minutes, showing fluctuations. Each color represents the junction tracked during a time bracket of 3 minutes, in the following order: black (0–2 min.), blue (3–5 min.), cyan (6–8 min.), green (9–11 min.), yellow (12–15 min.), red (16–18 min.), magenta (19–21 min.). (B) The mean square displacement was measured for the center of mass of each junction in n = 84 junctions (time-averaging with non-overlapping segments). The red line corresponds to the best fit (Kaleidagraph) with an α exponent of 1.14. The black dashed line corresponds to the resolution limit. For junction tracking, E-cadherin-GFP expressing cells were imaged typically every 30 s to 2 min, for 30 to 60 minutes at 37°C in DMEM medium complemented with 10% FBS and 10 mM Hepes using an IX71 Olympus microscope driven by Metamorph with 63X or 100X objectives. Fluorescent junctions were sequentially followed with a home-made Matlab routine: at each time point, junctions were manually outlined, and each point in the common segments was automatically attributed to its corresponding perpendicular counterpart from the previous time point. This Fig illustrates that movements of junctions are not purely convective, but contain a component that fluctuates. Although a local directive movement, at a longer time rate, exists for each junction, these directional movements are not correlated between junctions: it does not correspond to a global deformation field (see S2 Movie). (C) Overlay of 2 subsequent images of cell junctions separated by 10 minutes. The first image is represented in green, the second in red. No net displacement is visible here due to fluctuations in direction. Then, junctions are quasi immobile at a time scale (10 min) where molecular components of adherens junctions are entirely renewed (junction residence times between 30 s and 240 s for actin, E-cadherin and p120-catenin from our FRAP results). That suggests the existence of regulatory mechanisms correcting in permanence (at short time scales) the force imbalance at junctions.
(PDF)

S2 Fig E-cadherin turnover rate k_off_ as measured by FRAP at diffraction-limited spots in confluent layer of MDCK cells. (A) E-cadherin-GFP is bleached in different junctions. Images taken from S1 Movie show the fluorescence before bleaching, 1 second, 5 minutes or 15 minutes after bleaching. (B) An example of FRAP curves is shown. Experimental data (blue line) are fit with an exponential model (red line) typical of exchange reactions. K_off_ parameter is extracted from those fits for each curve.
(PDF)

S3 Fig *adherens* junctions relax to their initial position after traction is released. Merged images show adherens junction position at different times. a) before (green) and after 5 minutes of traction (red) with a pipette, in the direction of the arrow. b) After 5 minutes of traction (green) and 10 minutes after releasing the traction (red). The total net displacement is represented in c): the position after relaxation (red) matches the pre-traction position (green).
(PDF)

S4 Fig E-cadherin levels do not increase in junctions perturbed by tractions with pipettes. (A) Images before (top) and 30 minutes after beginning applying the traction (bottom). Direction of the traction applied is shown by a red arrow. (B) Fluorescence in the junction, normalized by the average fluorescence in the image, is measured before or while the traction is applied. Ratios distribution (n = 10) is represented in a dot plot. The red line represents the median of the distribution.
(PDF)

S5 Fig E-cadherin dynamics are symmetrical in position-clamped micro-pipette traction experiments. (A) Mechanical perturbation was carried out using glass micropipettes as described in Materials & Methods, by pulling on MDCK-EcadGFP cells in the vicinity of a junctions made with MDCK-cadDsRed expresing cells. Dual-colour FRAP was performed as described in Materials & Methods once junctions had reached a new stable position. (B) Ratios between E-cadherin turnover rates (k_off_) taken on each side of junctions under increased tension (red, N = 8) were close to one, and significantly similar (p = 0.36) to those measured in the absence of additional tension (blue, N = 13). The box plot represents quartile distribution.
(PDF)

S6 Fig In junctions pulled with pipettes, FRAP profiles do not widen with time. (A) Fluorescence profiles were measured in MDCK-Ecadh-GFP along the junctions at different times after photobleaching. Average profiles (n = 10) are represented: 0.6s (blue squares), 1 minute (red triangles) or 5 minutes (green circles). Data were fit with a gaussian model (plain lines), and the width (sigma) was extracted from these fits (B). Error bars represent the standard error of the mean. (C) A situation where E-cadherin dynamics is limited by first-order exchange with heterogeneous rates along the x position in the junctions was numerically simulated. Different time points after the beginning of relaxation are represented: 0s (black curve), 100s (red), 300s (green). The dissociation rate was kept constant from x = -4 to x = -1, and increased linearly from x = -1 to x = 1.5. The width was evaluated from Gaussian fits (D).
(PDF)

S7 Fig In cells adjacent to RhoA-injected cells, turnover still follows a space-independent first-order recovery. Fluorescence profiles were measured at different times after photobleaching in MDCK-Ecadh-DsRed cells adjacent to injected to RhoA-injected MDCK-Ecadh-GFP cells (N = 15). Profiles were fit with a gaussian function indicating a width σ that remained constant with time. Error bars: standard error to the mean.
(PDF)

S1 Movie An example of FRAP experiments. 2-photon photobleaching is achieved in diffraction-limited spots (arrows) in junctions between MDCK cells expressing E-cadh-GFP. Total duration: 15 minutes. Image size: 45.5 x 35 μm.
(AVI)

S2 Movie Mechanical fluctuations of cell-cell junctions. MDCK-E-cadh GFP were imaged for 20 hours (1 image per hour).
(AVI)
