# Supplementary figures and images for: Mechanosensitive Adaptation of E-Cadherin Turnover across adherens Junctions
Source: PLoS One. 2015 Jun 5;10(6):e0128281. doi: 10.1371/journal.pone.0128281 (PMC4457789; doi:10.1371/journal.pone.0128281)

**A****example 1**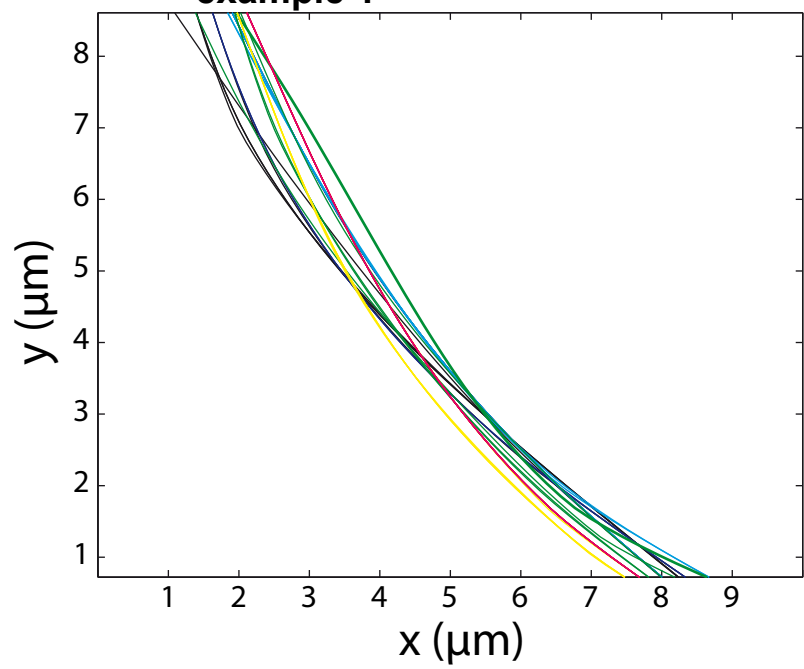**example 2**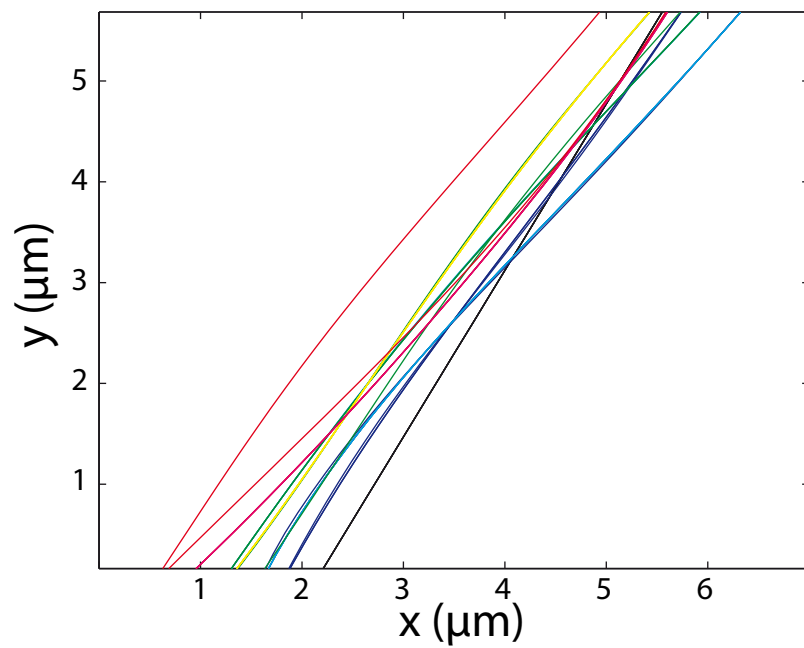**B**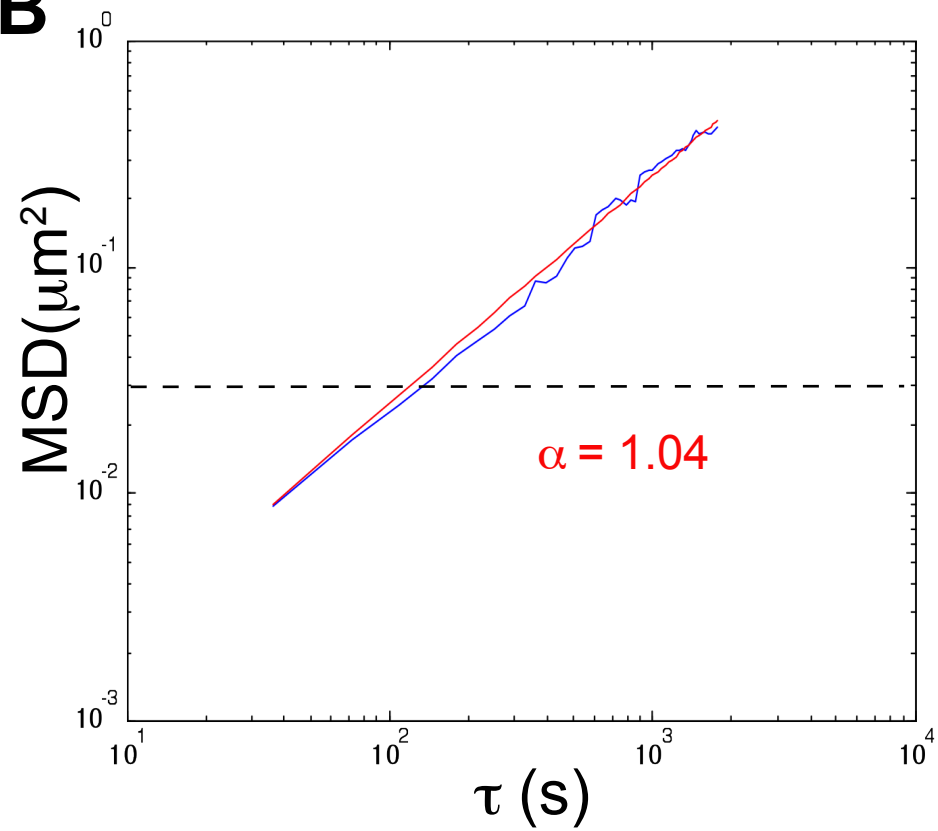**C**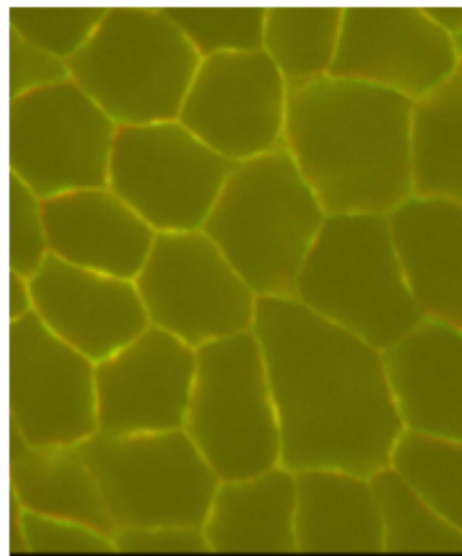

Supplement: S1 Fig — (A) 2 examples of cell junction trajectories over 20 minutes, showing fluctuations. Each color represents the junction tracked during a time bracket of 3 minutes, in the following order: black (0–2 min.), blue (3–5 min.), cyan (6–8 min.), green (9–11 min.), yellow (12–15 min.), red (16–18 min.), magenta (19–21 min.). (B) The mean square displacement was measured for the center of mass of each junction in n = 84 junctions (time-averaging with non-overlapping segments). The red line corresponds to the best fit (Kaleidagraph) with an α exponent of 1.14. The black dashed line corresponds to the resolution limit. For junction tracking, E-cadherin-GFP expressing cells were imaged typically every 30 s to 2 min, for 30 to 60 minutes at 37°C in DMEM medium complemented with 10% FBS and 10 mM Hepes using an IX71 Olympus microscope driven by Metamorph with 63X or 100X objectives. Fluorescent junctions were sequentially followed with a home-made Matlab routine: at each time point, junctions were manually outlined, and each point in the common segments was automatically attributed to its corresponding perpendicular counterpart from the previous time point. This Fig illustrates that movements of junctions are not purely convective, but contain a component that fluctuates. Although a local directive movement, at a longer time rate, exists for each junction, these directional movements are not correlated between junctions: it does not correspond to a global deformation field (see S2 Movie). (C) Overlay of 2 subsequent images of cell junctions separated by 10 minutes. The first image is represented in green, the second in red. No net displacement is visible here due to fluctuations in direction. Then, junctions are quasi immobile at a time scale (10 min) where molecular components of adherens junctions are entirely renewed (junction residence times between 30 s and 240 s for actin, E-cadherin and p120-catenin from our FRAP results). That suggests the existence of regulatory mechanis [file pone.0128281.s001.pdf]

**A**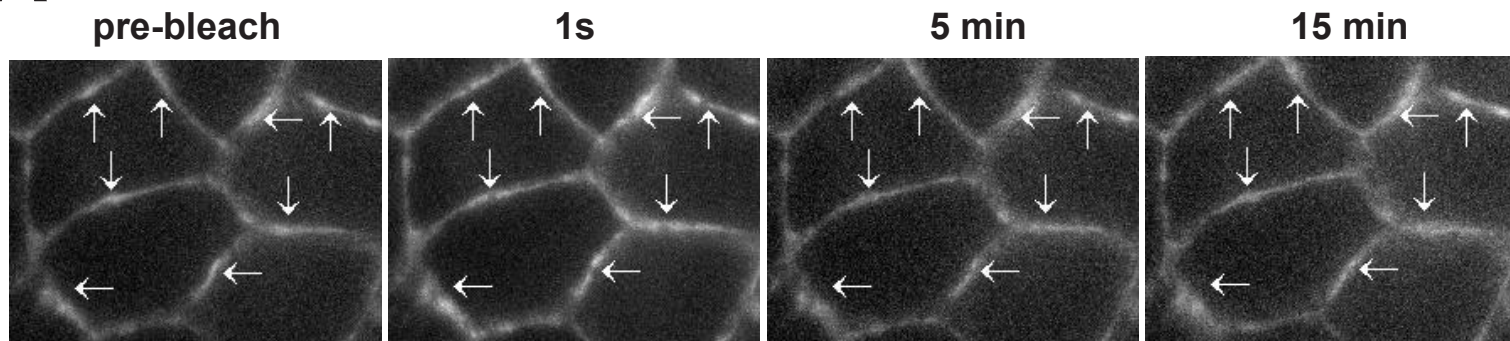**B**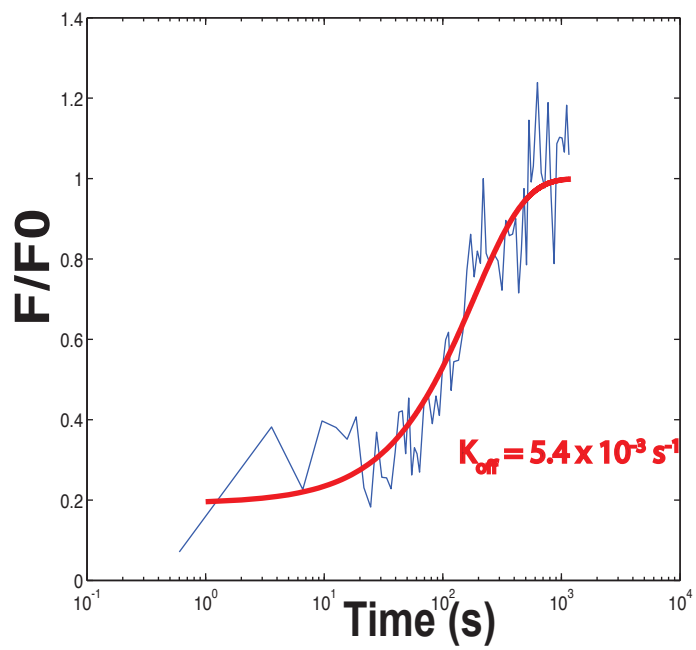

Supplement: S2 Fig — (A) E-cadherin-GFP is bleached in different junctions. Images taken from S1 Movie show the fluorescence before bleaching, 1 second, 5 minutes or 15 minutes after bleaching. (B) An example of FRAP curves is shown. Experimental data (blue line) are fit with an exponential model (red line) typical of exchange reactions. Koff parameter is extracted from those fits for each curve. (PDF) [file pone.0128281.s002.pdf]

**A**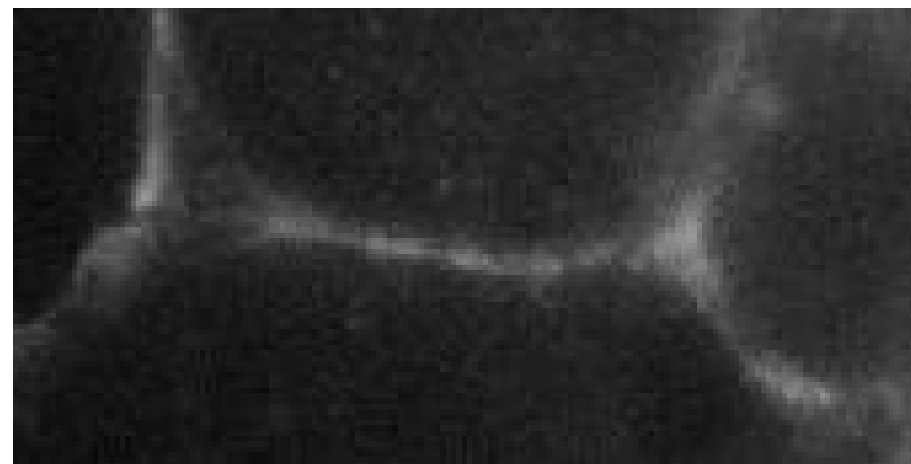

pulling with  
pipette

20 min

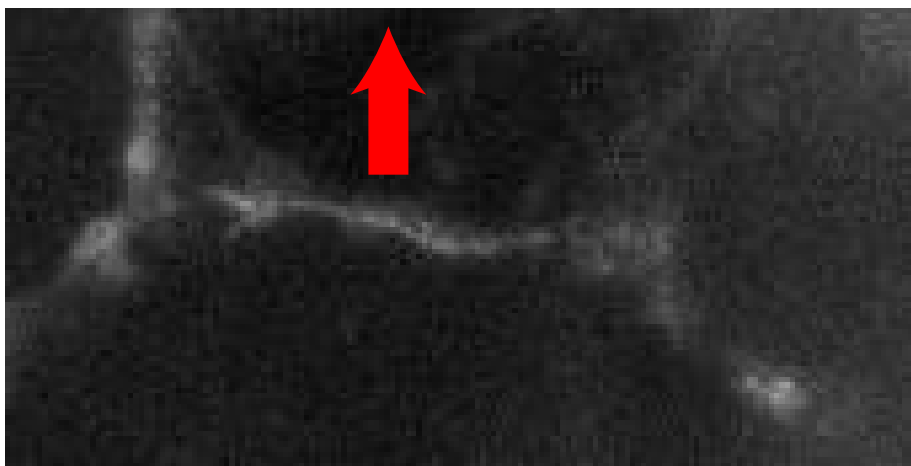**B**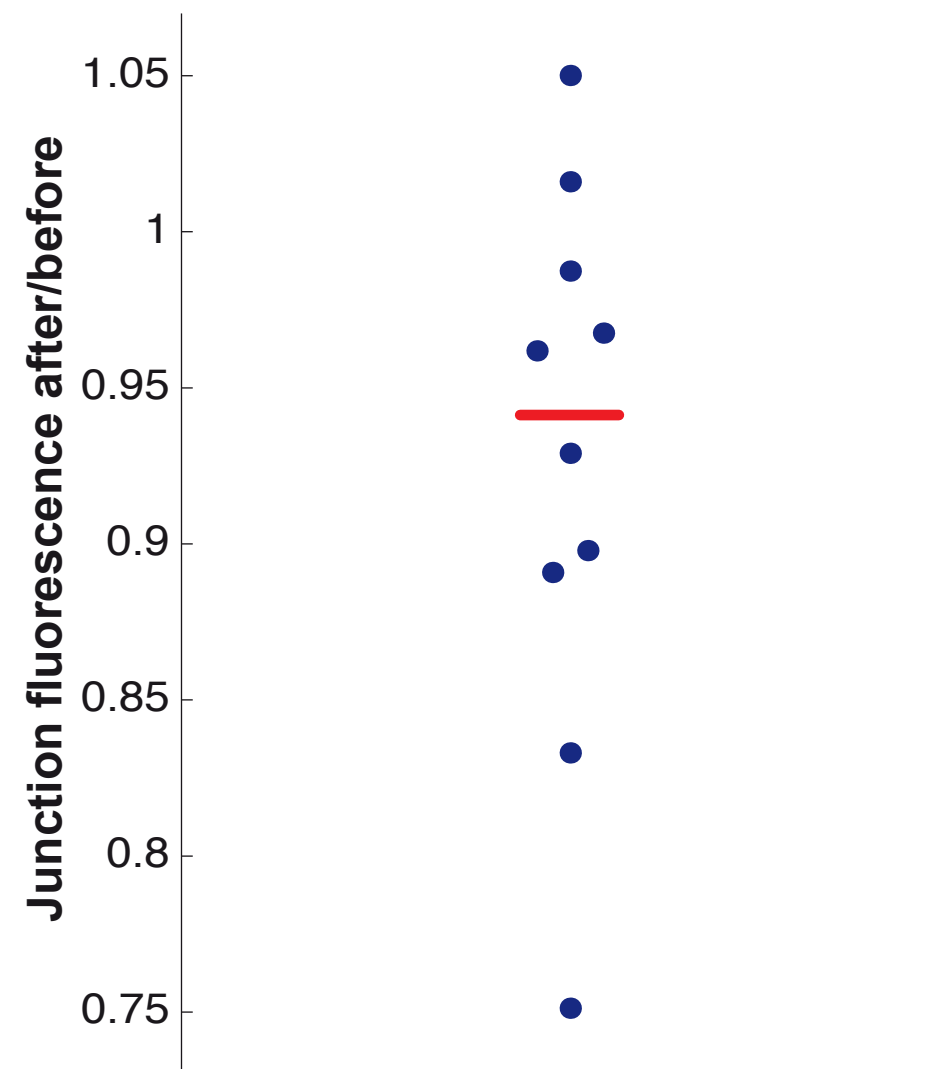

Supplement: S4 Fig — (A) Images before (top) and 30 minutes after beginning applying the traction (bottom). Direction of the traction applied is shown by a red arrow. (B) Fluorescence in the junction, normalized by the average fluorescence in the image, is measured before or while the traction is applied. Ratios distribution (n = 10) is represented in a dot plot. The red line represents the median of the distribution. (PDF) [file pone.0128281.s004.pdf]

**A**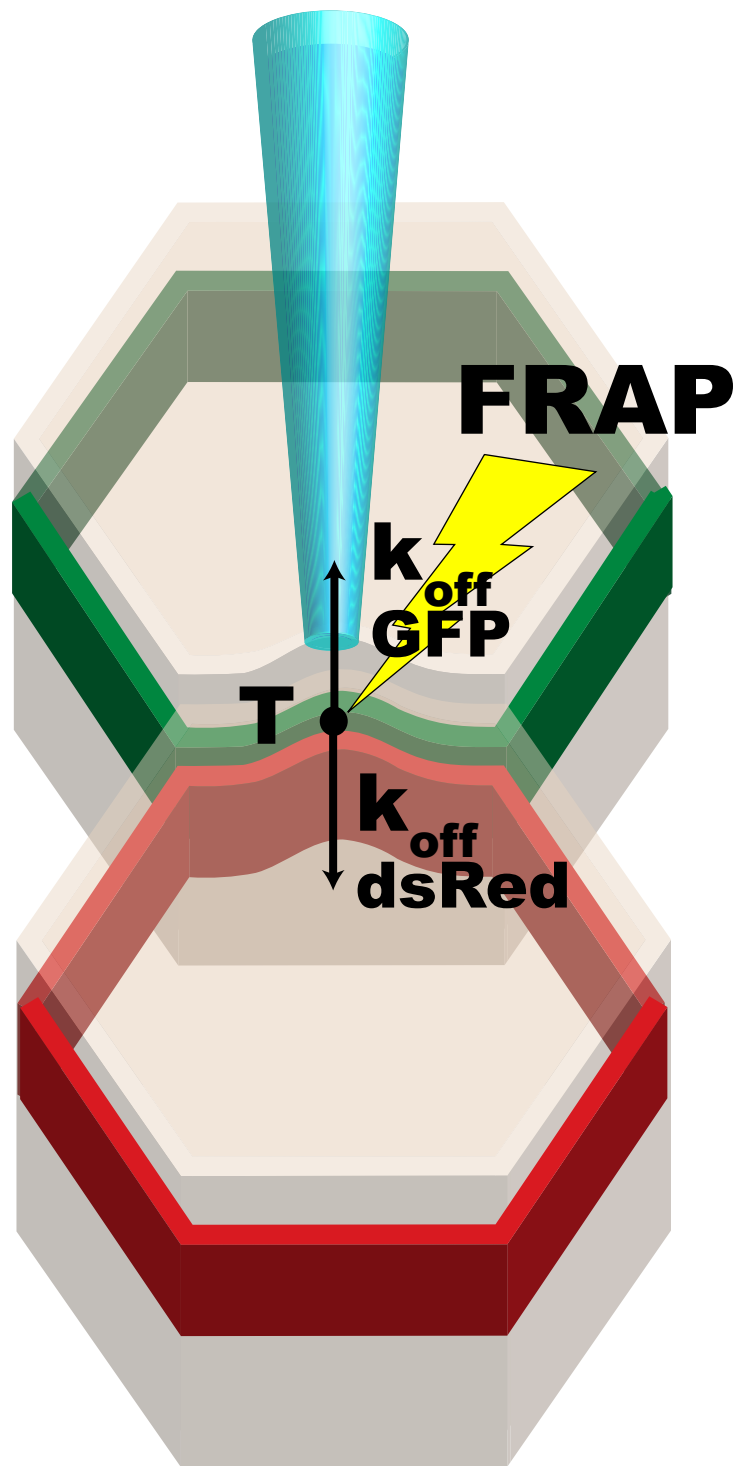**B**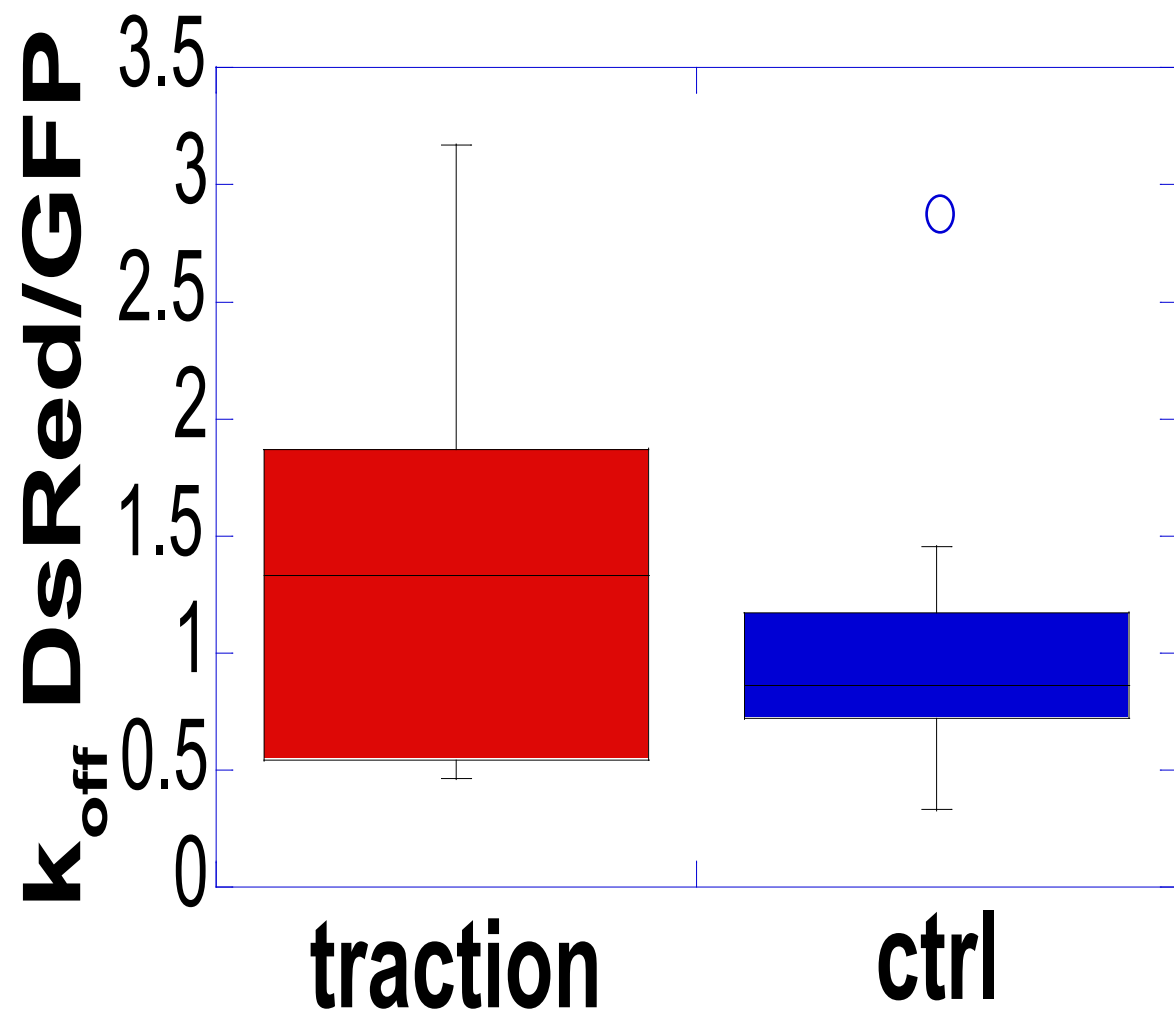

Supplement: S5 Fig — (A) Mechanical perturbation was carried out using glass micropipettes as described in Materials & Methods, by pulling on MDCK-EcadGFP cells in the vicinity of a junctions made with MDCK-cadDsRed expresing cells. Dual-colour FRAP was performed as described in Materials & Methods once junctions had reached a new stable position. (B) Ratios between E-cadherin turnover rates (koff) taken on each side of junctions under increased tension (red, N = 8) were close to one, and significantly similar (p = 0.36) to those measured in the absence of additional tension (blue, N = 13). The box plot represents quartile distribution. (PDF) [file pone.0128281.s005.pdf]

**A**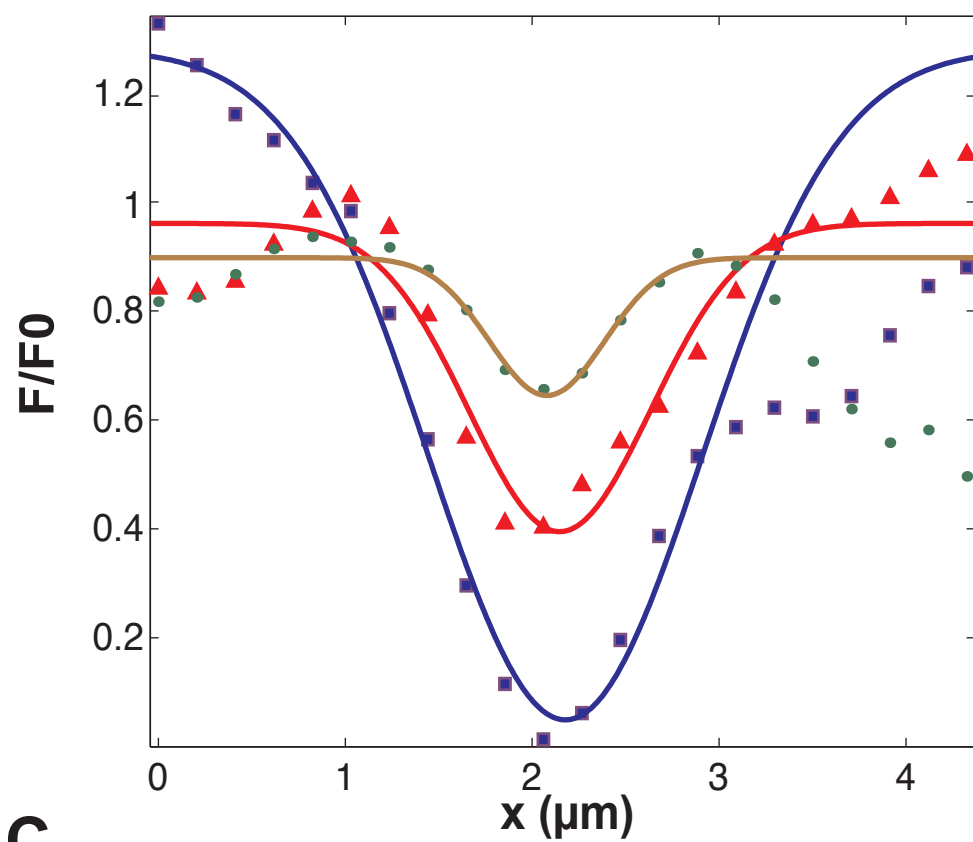**B**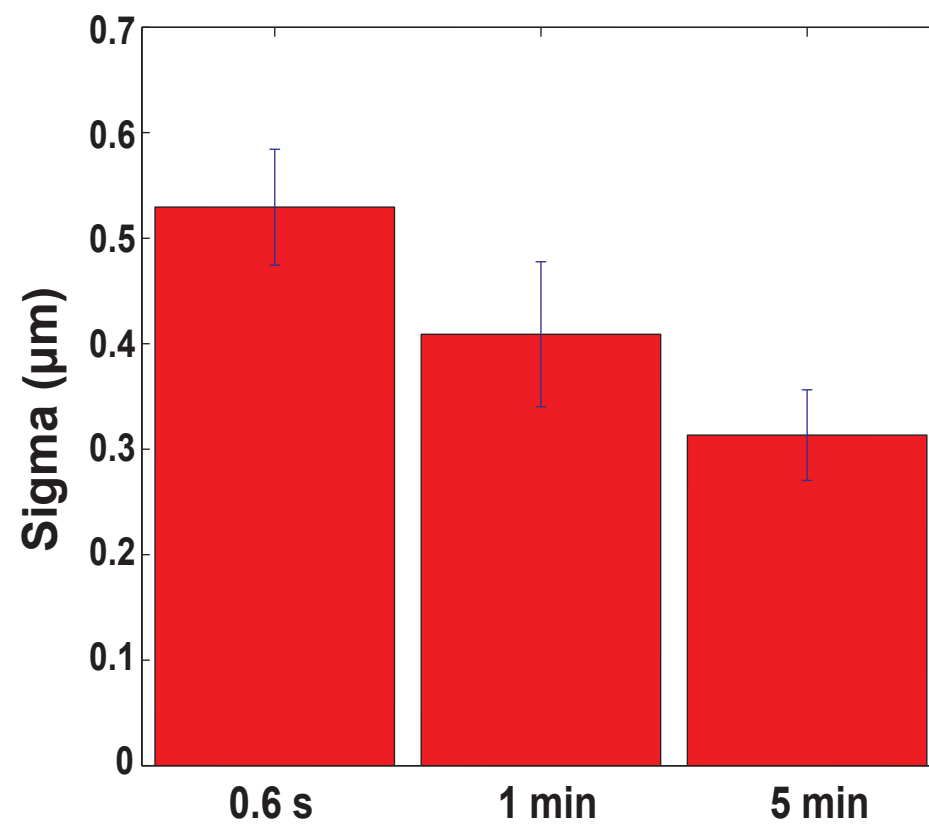**C**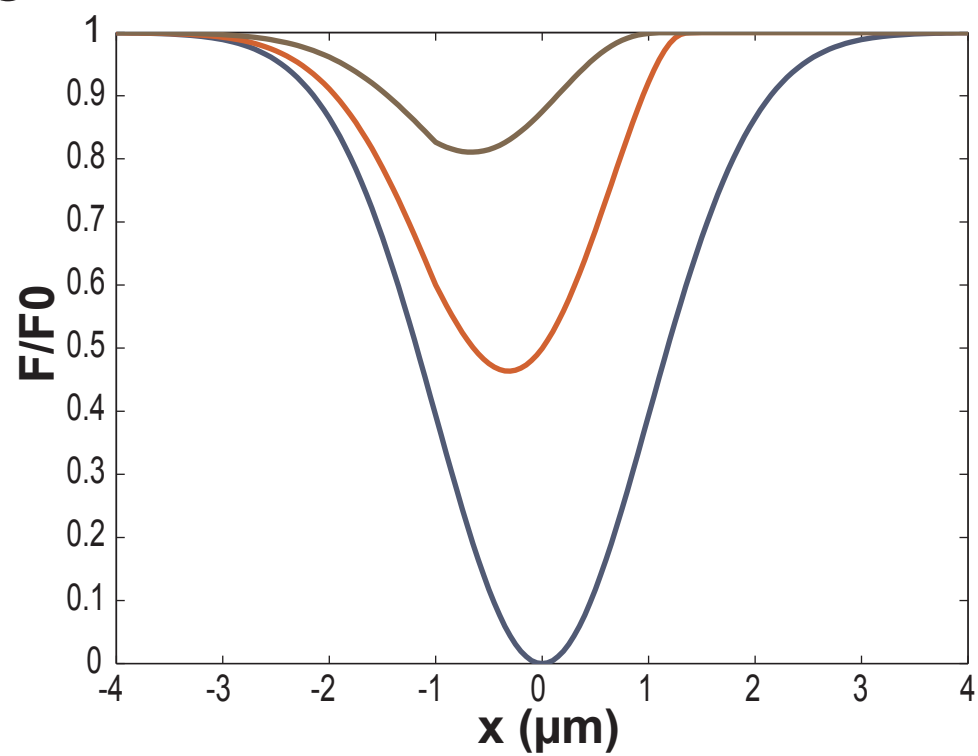**D**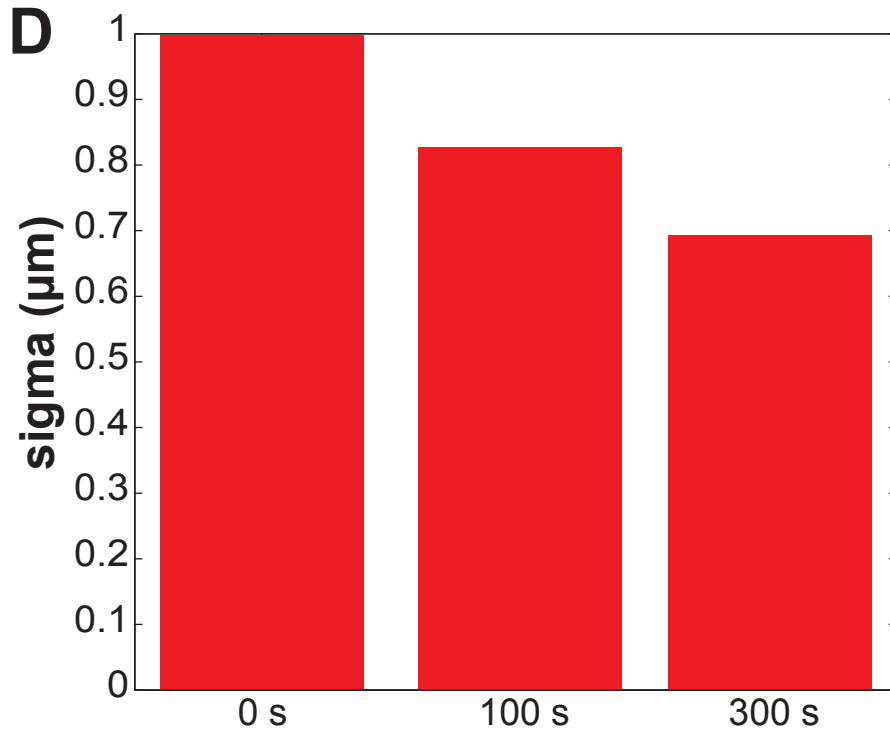

Supplement: S6 Fig — (A) Fluorescence profiles were measured in MDCK-Ecadh-GFP along the junctions at different times after photobleaching. Average profiles (n = 10) are represented: 0.6s (blue squares), 1 minute (red triangles) or 5 minutes (green circles). Data were fit with a gaussian model (plain lines), and the width (sigma) was extracted from these fits (B). Error bars represent the standard error of the mean. (C) A situation where E-cadherin dynamics is limited by first-order exchange with heterogeneous rates along the x position in the junctions was numerically simulated. Different time points after the beginning of relaxation are represented: 0s (black curve), 100s (red), 300s (green). The dissociation rate was kept constant from x = -4 to x = -1, and increased linearly from x = -1 to x = 1.5. The width was evaluated from Gaussian fits (D). (PDF) [file pone.0128281.s006.pdf]

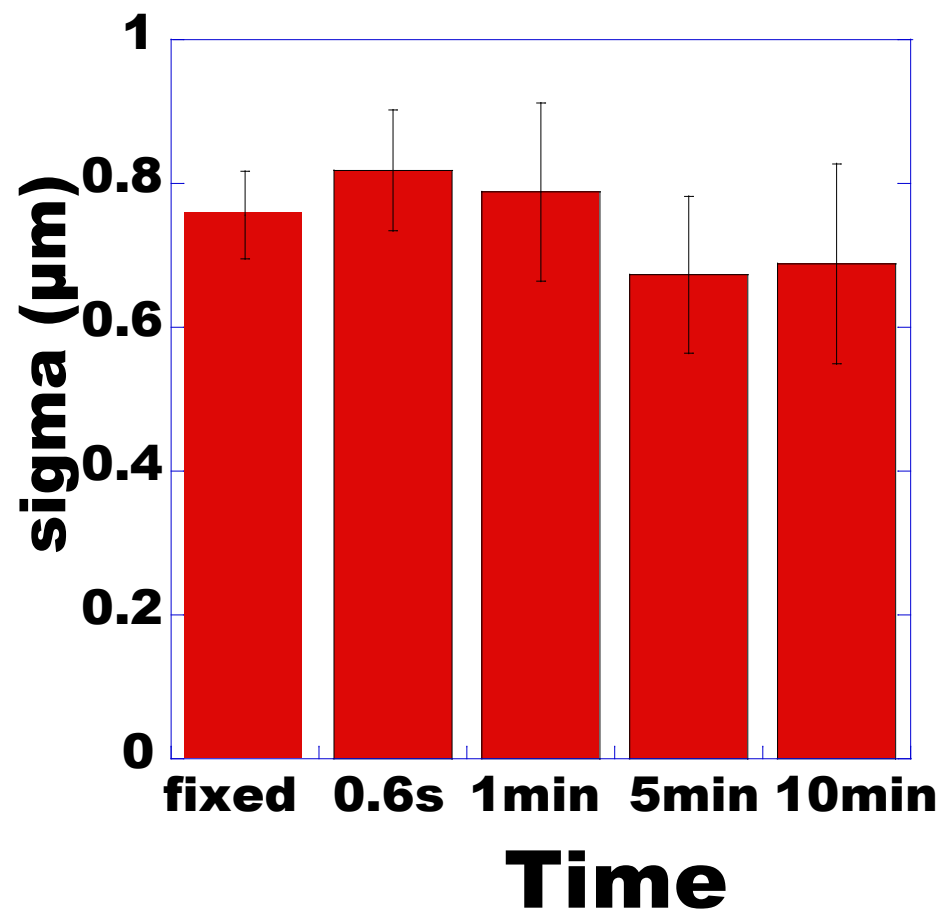

Supplement: S7 Fig — Fluorescence profiles were measured at different times after photobleaching in MDCK-Ecadh-DsRed cells adjacent to injected to RhoA-injected MDCK-Ecadh-GFP cells (N = 15). Profiles were fit with a gaussian function indicating a width σ that remained constant with time. Error bars: standard error to the mean. (PDF) [file pone.0128281.s007.pdf]
