# Supplementary material for: Mechanosensitive Adaptation of E-Cadherin Turnover across adherens Junctions
Source: PLoS One. 2015 Jun 5;10(6):e0128281. doi: 10.1371/journal.pone.0128281 (PMC4457789; doi:10.1371/journal.pone.0128281)
Supplement: S3 Fig — Merged images show adherens junction position at different times. a) before (green) and after 5 minutes of traction (red) with a pipette, in the direction of the arrow. b) After 5 minutes of traction (green) and 10 minutes after releasing the traction (red). The total net displacement is represented in c): the position after relaxation (red) matches the pre-traction position (green). (PDF) [file pone.0128281.s003.pdf]

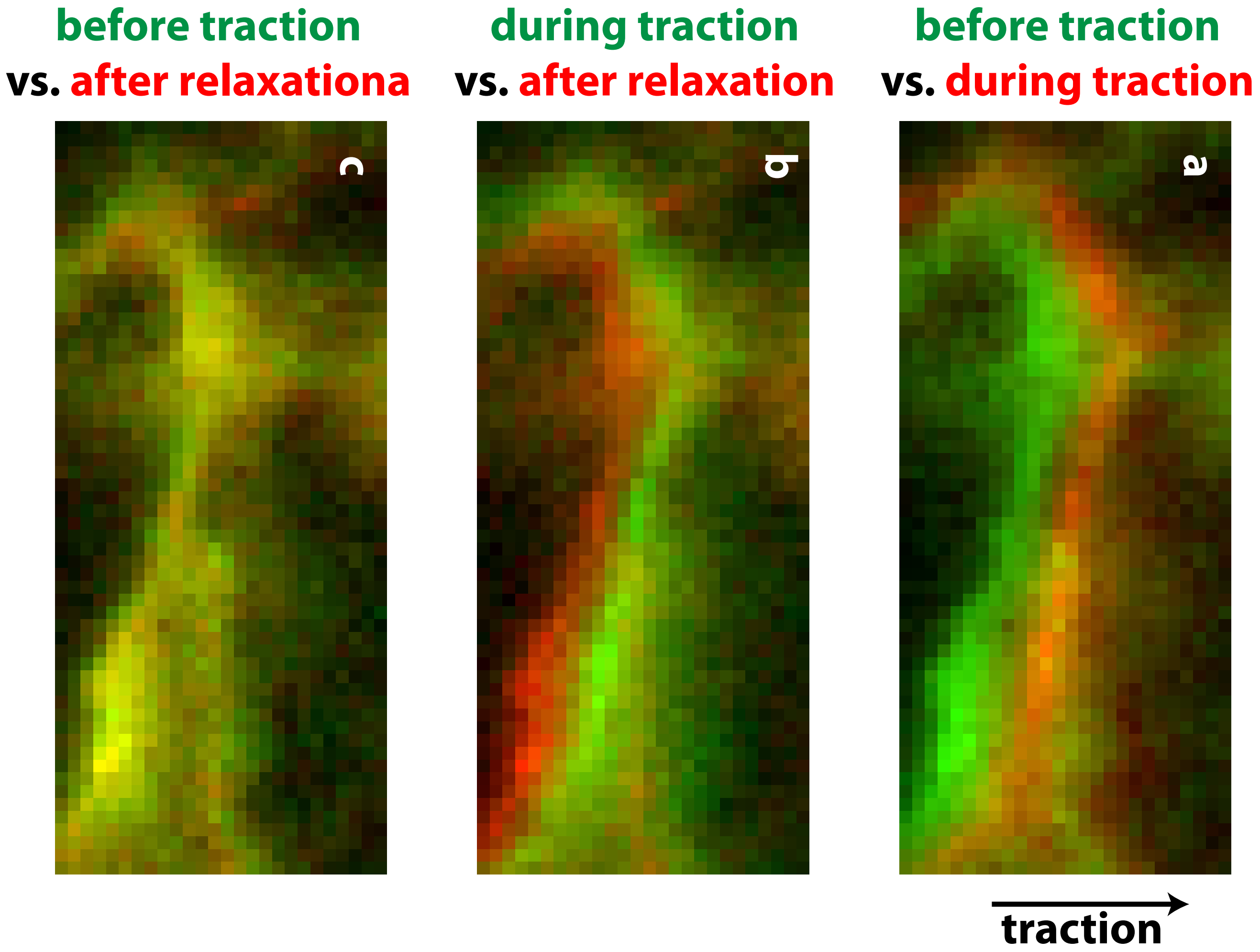
before traction
vs. after relaxationa
during traction
vs. after relaxation
before traction
vs. during traction
c
b
a
traction
